# Luminescence in the fluoride-containing phosphate-based glasses: A possible origin of their high resistance to nanosecond pulse laser-induced damage

**DOI:** 10.1038/srep08593

**Published:** 2015-02-26

**Authors:** Pengfei Wang, Min Lu, Fei Gao, Haitao Guo, Yantao Xu, Chaoqi Hou, Zhiwei Zhou, Bo Peng

**Affiliations:** 1 State Key Laboratory of Transient Optics and Photonics, Xi'an Institute of Optics and Precision Mechanics, Chinese Academy of Sciences, 710119, Xi'an, P. R. China; 2 Institute of Nuclear and New Energy Technology, Tsinghua University, Beijing100084, P. R. China; 3 Institute of Advanced Materials, Nanjing University of Posts & Telecommunications, Nanjing, 210023, P. R. China

## Abstract

Fusion power offers the prospect of an almost inexhaustible source of energy for future generations. It was reported that fusion fuel gains exceeding unity on the National Ignition Facility (NIF) were achieved, but so far great deal of scientific and engineering challenges have to be overcome for realizing fusion power generation. There is a bottleneck for color-separation gratings in NIF and other similar inertial confinement fusion (ICF) lasers. Here we show a series of high performance phosphate-based glasses that can transmit the third harmonic frequency (3ω) laser light with high efficiency meanwhile filter the fundamental (1ω) and the second harmonic frequency (2ω) laser lights through direct absorption, and especially they exhibit excellent damage threshold induced by nanosecond pulse laser compared with that of the fused silica used in NIF. Yellowish-orange fluorescence emits during the laser-material interaction process, and it can be tailored through regulating the glass structure. Study on its structural origin suggests that the fluorescence emission is a key factor that conduces to the high laser-induced damage resistance of these glasses. The results also indicated the feasibility of utilizing these high performance glasses in novel color separation optics, allowing novel design for the final optics assembly in ICF lasers.

The attraction of perspective inexhaustible clean energy source from nuclear fusion has stimulated worldwide intensive research on giant lasers similar to the NIF for many years. The US NIF is thought to be the first step on the way to achieve potential unlimited fusion energy through laser-driven controlled inertial confinement fusion^1^,^2^. A most promising fusion shot on the NIF took place on last September^3^, followed by a latest report on achievement of fusion fuel gains exceeding unity on the NIF^4^. But the existing unexpected technical problems still forced the ICF researchers to accept that the ignition is still a long way off to carry the far-off promise of clean energy^3^,^5^,^6^. Meanwhile, the NIF fusion researchers are trying to identify some of these obstacles and addressing the challenges to getting to ignition^7^.

It is known to fusion scientists that the color phase separation in final optics assembly (FOA) plays a key role in high-energy and high-peak-power laser systems like the NIF for color management at target that primarily controls the 3ω laser beam conditioning. In ICF laser systems around the world, most of the glass lasers employ non-linear frequency conversion for converting fundamental wave to its second harmonic and third harmonic lights^8^. Inevitably, the remnant energy of 1ω and 2ω frequency lights remains in optical path after frequency conversion. The unconverted lights as enter fusion target are harmful to ICF experiments, and they should be filtered out as clean as possible. At present, color phase separation in the NIF is realized through color-separation gratings (CSGs) in fused silica substrates to transmit 3ω light while redirecting unconverted 1ω and 2ω lights away from the fusion target^9^,^10^,^11^. However, in fact both the unconverted 1ω and 2ω lights will continuously propagate into the target chamber and have a resultant offset focus near the fusion target. The envelope of unconverted light extends out from target chamber center, potentially affecting the target back lighter structures and diagnostics^12^,^13^. Besides these technical problems, the diffractive optical elements used in the FOA that are made from fused silica glass are always damaged by stimulated Brillouin scattering under high laser fluence^14^. In addition, to fabricate the large aperture (43 cm × 43 cm) optics in the FOA, such as color-separation gratings and silica wedge focus lens, must suffer most harsh processing techniques^11^ and therefore is time consuming and expensive. Now, these related problems due to existing color phase separation technique, together with the limit of improving the laser damage resistance of the fused silica glasses, has become one of the most significant bottlenecks to further increase the output fluence of ICF laser systems.

As early as in 1999, the NIF council had pointed out that the catastrophic damage of fused silica at high fluence in a vacuum fundamentally calls into the question whether fused silica can ever go the distance to reliable operation at full NIF fluence or above^15^. Thus they recommended that the effort should be undertaken to develop some better materials for operation at 8–9 joules/cm^2^, and to determine under what conditions existing materials can operate reliably at such levels. And the materials should be developed with an eye towards replacing or protecting the silica optical elements between the DKDP crystal and the target. To develop replacement options for fused silica will allow more novel designs for the FOA^15^.

There are a variety of conceptual candidates to replace fused silica, such as fluoride crystals, fluoride glasses, and others^15^, among which the fluoride-phosphate glasses are verified to the potentially good UV transmitting materials^16^. On the other hand, because of the increasing demands of the high performance UV transmitting materials to be applied for lens system in UV microlithography equipments, excimer laser systems and other special UV optics, since the early 1990's, researchers has already begin systematic investigations^17^,^18^,^19^,^20^,^21^,^22^,^23^,^24^,^25^,^26^,^27^,^28^,^29^,^30^ on the optical performance of various fluoride-phosphate and other phosphate-based glasses due to the existence of intrinsic defects, extrinsic impurity ions or under the irradiations of UV-lamp, excimer lasers, in order to explore the applicability of these glass materials. Recently, we also noticed that a kind of ultraviolet transmitting near infrared cut filter boron-containing phosphate-based glasses doped with CuO as absorptive agent^31^, which had a high UV transmittance and a low near infrared transmittance were proposed for the solid-state imaging elements or use in high power lasers for lithography, laser processing and possible use in laser fusion, but there is no report of their laser-induced damage property, which is a necessity for potential use in the ICF laser systems.

In order to solve the above problem relating to the existing color phase separation technique and meanwhile to explore novel designs for the FOA in ICF lasers, here we reported a series of new ultraviolet transmitting, 1ω and 2ω absorptive fluoride-containing phosphate-based glasses, which are not only highly transparent to the 3ω laser wavelength but selectively intense absorptive to the 1ω and 2ω laser lights, therefore 1ω and 2ω laser lights can be more easily separated through direct absorption method, which is completely different from the present color phase separation technique using the color separation gratings. More worthy of mention is the proposed phosphate-based glasses also maintain excellent performance in resistance to nanosecond pulse laser-induced damage, for which the laser-induced surface damage threshold and growth damage threshold at 351 nm (8 ns@3ω) is testified to be as high as that of fused silica and even higher. At relative lower laser beam fluence the new phosphate-based glass has a comparable damage growth rate with that of the fused silica, and under larger beam fluence irradiations its damage grows faster than fused silica. It may suggest that the 3ω transparent fluoride-containing phosphate-based glass should have high comprehensive resistance to nanosecond pulse laser-induced damage, which is better than or at least compariable with that of the fused silica under the same boundary conditions. From the systematic analysis in this work, it suggests that their high 3ω laser-induced damage threshold is attributed to the special laser-induced fluorescence character that was verified through contrast experiments can be realized through special tailoring on the glass structure.

## Results

### Spectroscopic property and laser-induced damage thresholds of the series of phosphate-based glasses

**Figure 1:**
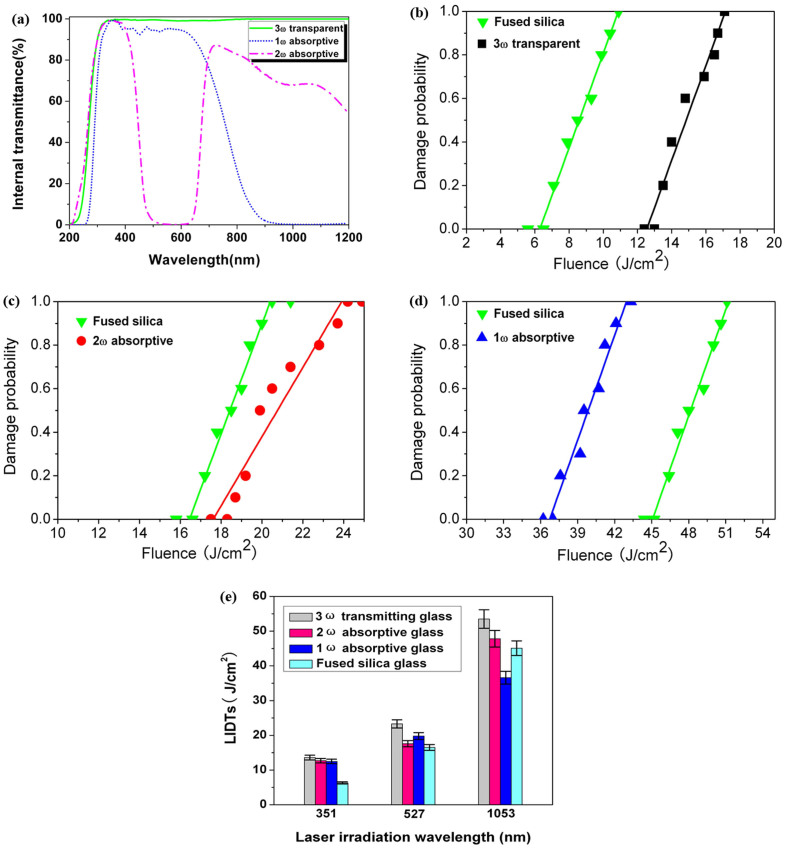
Spectroscopic and damage resistance properties of the series of phosphate-based glasses with specially tailored structure. (a) Internal transmission spectra. Each sample has a path length of 10 mm, both surfaces uncoated. (b)–(e) Zero damage probability of the series of phosphate-based glasses and fused silica (with a thickness of 7 mm, uncoated) under different laser irradiation wavelengths: (b) 3ω transparent glass and fused silica at 351 nm, (c) 2ω absorptive glass and fused silica at 527 nm, (d) 1ω absorptive glass and fused silica at 1053 nm, and (e) comparison of the laser-induced damage threshold between the phosphate-based glasses and fused silica glass under three laser irradiation wavelengths.

Fig. 1 shows the spectroscopic and laser-induced damage probability of the series of phosphate-based glasses with specially tailored structure. As seen from Fig. 1a, the glasses maintain high 3ω (351 nm) internal transmittance (τ_351 nm_). The τ_351 nm_ reaches up to 99.5% for the 3ω transparent glass, and it is about 98.6% and 99.2% for the 1ω and 2ω absorptive glass, respectively. The internal transmittance at 1053 nm (τ_1053 nm_) is as low as 0.14% for the 1ω absorptive glass and the internal transmittance at 527 nm (τ_527 nm_) is about 0.51% for the 2ω absorptive glass.

Besides the feature of intense absorption towards 1ω or 2ω laser lights, these phosphate-based glasses also possess excellent performance of damage resistance to nanosecond pulse laser irradiation. As shown in Fig. 1b, the LIDT values (8 ns@3ω) corresponding to zero damage probability of the 3ω transparent glass at 351 nm laser wavelength is 13.5 J/cm^2^, but it is 6.3 J/cm^2^ for the fused silica. The 2ω absorptive glass has a LIDT value of 17.6 J/cm^2^ at 527 nm, and it is 16.5 J/cm^2^ for the fused silica (Fig. 1c). As can be seen from Fig. 1d, only at the wavelength of 1053 nm, the LIDT value of the 1ω absorptive glass is (36.6 J/cm^2^) is relatively lower than that of the fused silica, 45.1 J/cm^2^.

The contrast LIDT test (8 ns@3ω) results in Fig. 1e indicate that, under the irradiation of 351 nm pulse laser, the LIDT value corresponding to zero damage probability of the series of phosphate-based glasses on the rear surface decreases in the order of the 3ω transparent glass (13.5 J/cm^2^), the 2ω absorptive glass (12.7 J/cm^2^) and the 1ω (12.5 J/cm^2^) absorptive glass, which is due to the increasing absorption coefficient at the 3ω wavelength. And each value is nearly twice that of the fused silica (6.3 J/cm^2^). As irradiated by 527 nm nanosecond pulse laser, it is viewed that the LIDT values of these glasses (23.3 J/cm^2^, 17.6 J/cm^2^ and 19.8 J/cm^2^ for the 3ω transparent, 2ω and 1ω absorptive glasses, respectively) are also higher than that of fused silica (16.5 J/cm^2^). Furthermore, the zero probability LIDT at 1053 nm increases to 53.5 J/cm^2^ and 47.8 J/cm^2^ for the 3ω transparent glass and the 2ω absorptive glass, respectively. Generally, the more intense absorption of the material at the laser wavelength, the easier it will be damaged by the laser due to more serious thermal effects. Therefore, it is reasonable that the zero probability LIDT at 3ω wavelength is high because of the higher 3ω internal transmittance, i.e. lower absorption coefficient at 3ω wavelength. However, the series of glasses generally exhibit higher LIDT as compared with that of fused silica glass, except in the case of 1ω absorptive glass under 1053 nm laser irradiation.

### Laser-induced fluorescence (LIF) and its effect on the laser-induced damage threshold (LIDT)

**Figure 2:**
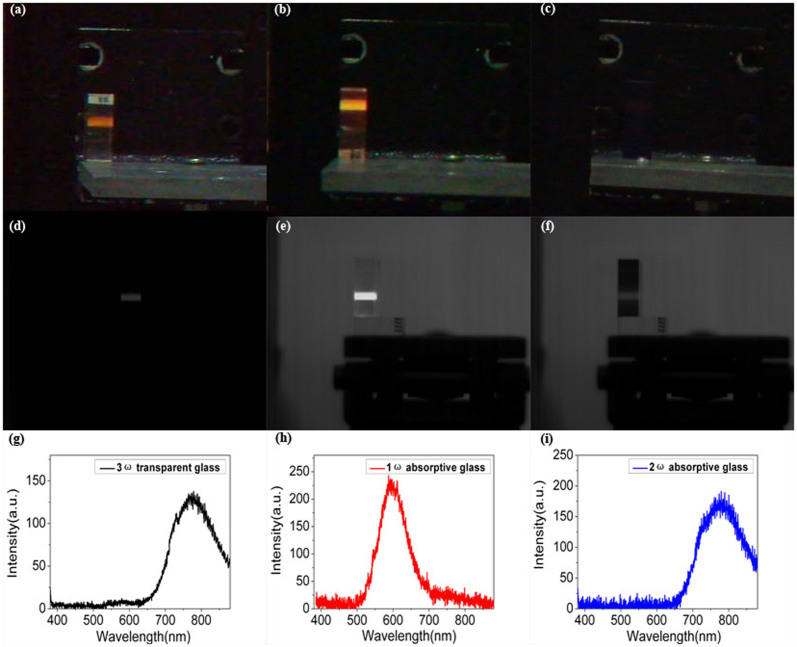
Laser-induced fluorescence phenomenon of the series of phosphate-based glasses under the 351 nm pulse laser irradiation. (a)–(c) Captured synchronously by a panchromatic camera, (d)–(f) Captured synchronously by a high speed monochrome camera for the 3ω transparent glass, 1ω absorptive and 2ω absorptive glass, respectively. (g)–(i) Laser-induced fluorescence spectra for the series of glasses.

More interesting, we occasionally found that the series of glasses display distinct fluorescent characteristics during their LIDT experiments. As shown in Fig. 2a–c, yellowish-orange laser-induced fluorescence are captured by the panchromatic camera, and the fluorescence intensity of their corresponding 8^th^ frame as indicated by the high speed monochrome camera photographs differs largely for these three glasses (Fig. 2d–f). By means of the high speed monochrome camera, the decay times of the laser-induced fluorescence are estimated to be as long as tens of millisecond and the estimated decay time of the 1ω absorptive glass is much longer than that of the 3ω transparent and 2ω absorptive glass. The corresponding laser-induced fluorescence spectra are shown in Fig. 2g–i. Under the 351 nm laser irradiation, one broad fluorescence band centered close to 600 nm is observed for the 1ω absorptive glass, and the fluorescence peak position shift to about 776 nm for both the 3ω transparent and the 2ω absorptive glass but their fluorescence intensity become relative weak.

**Figure 3:**
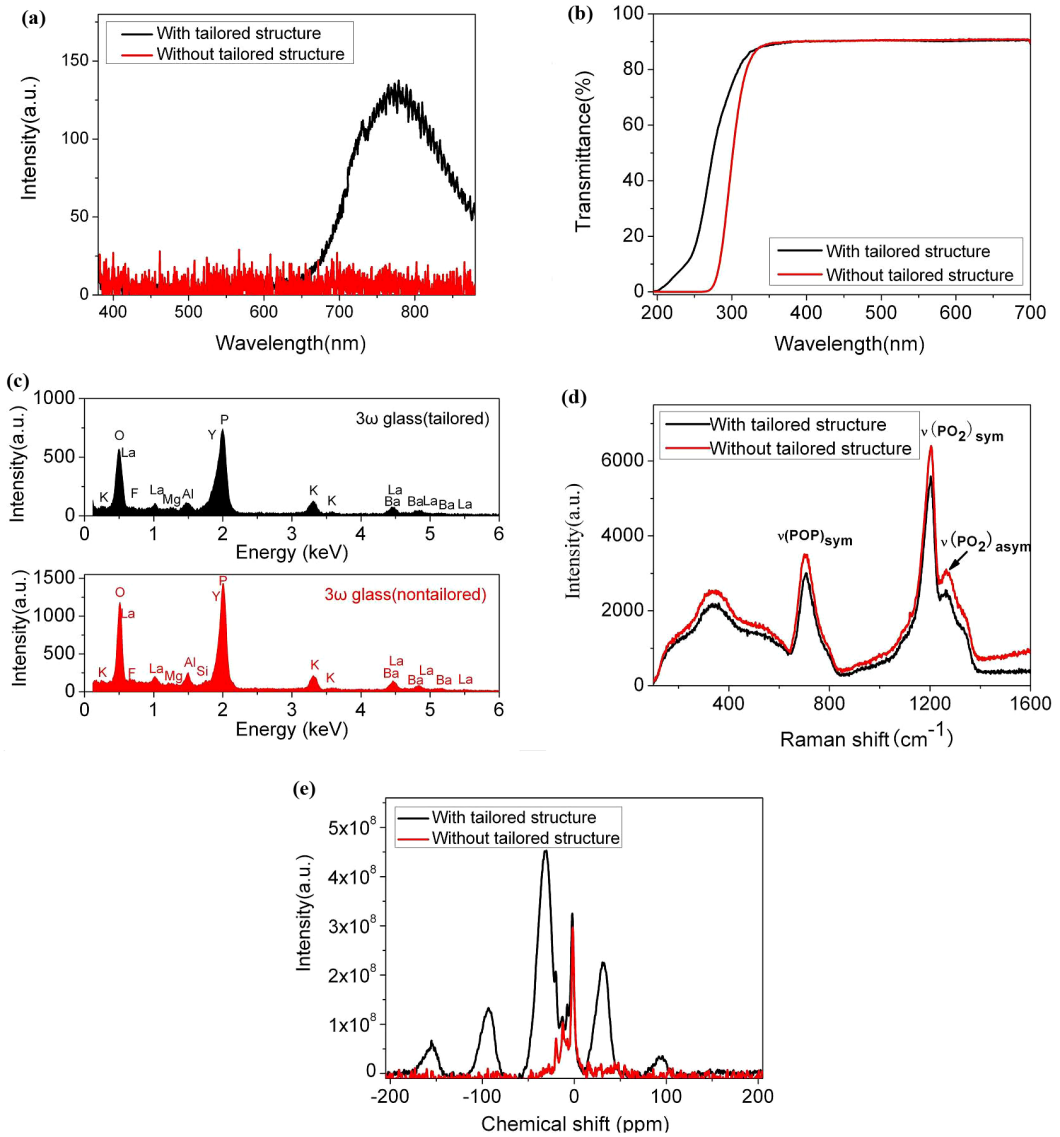
Contrast investigations on the 3ω transparent glasses with and without specially tailored structure. (a) Laser-induced fluorescence spectra, (b) The transmission spectra (with Fresnel reflection), (c) EDS spectra, (d) Raman spectra and (e) ^31^P nuclear magnetic resonance (NMR) spectra.

For further investigation on the interrelation between LIF and LIDT, first we have taken contrast experiments on the 3ω transparent glass that has no absorption towards 1ω or 2ω light. Fortunately, we found that the 3ω transparent glass which was prepared in an ambient air atmosphere did not show any visible fluorescence under the 351 nm laser shots, as indicated in Fig. 3a, but in clear contrast the laser-induced fluorescence is notable for the 3ω transparent glass prepared in a special reducing atmosphere. Accordingly, the former's LIDT at 351 nm (8 ns@3ω) was measured to be 8.4 J/cm^2^, which is much lower than that of the latter one (13.5 J/cm^2^). For easy comparison, hereafter the 3ω transparent glass prepared in the special reducing atmosphere is named as the 3ω transparent glass with tailored structure and the one melted in an ambient air atmosphere labeled as the 3ω transparent glass without tailored structure.

From the above significant change of LIDT with its different fluorescent character for the 3ω transparent glasses, it is clear to see that the fluorescence is an important factor contributing to the glasses' high laser-induced damage threshold. We further deduced that the striking decrease of the LIDT value, which depends on the glass's fluorescence character rather than the glass composition, most probably originates from their difference in the glass structure. It is clear that further research leading to a better understanding of the glass structure will continue to benefit development of the glasses with high LIDT values.

### Glass structure characterization and micro-defects analysis for the 3ω transparent glasses

What kind of difference in the structure or micro-defects inside the glasses will lead to the apparent contrast in the fluorescence character? Firstly, it is observed that the ultraviolet absorption edge of the former one shifts to the long wavelength side obviously even the transmittance at 351 nm changes very little (Fig. 3b). Because the reducing glass melting atmosphere suppressed the formation of Fe^3+^ impurity and defects in the glass that have absorption in the ultraviolet region. These are very small fluoride contents in these glasses, and the fluoride will be evaporated during the glas melting process. So we have examined the glass especially with respect to the fluoride concentration of the two as-prepared 3ω transparent glass samples with and without specially tailored structure by means of the energy dispersive spectrometer (EDS). The EDS spectra are shown in Fig. 3c and comparative analyses of the elemental composition in the two samples are presented in Table 1. As EDS is not very sensitive to the light elements with an atomic number smaller than boron (B), the lithium (Li) element is not found here. The EDS spectra of the as-prepared glasses still indicates the presence of the fluoride element in the glass after the long time duration at the high melting temperature (1200°C). As seen from Table 1, with regard to the fluoride content in the two undoped phosphate-based glass (3ω transparent glass), it is 4.88 wt% and 3.36 wt% for the glass that was melted in a closed iraurite crucible in a reducing atmosphere and the one in a silica crucible at the ambient air atmosphere, respectively. It is regarded that the closed melting conditions in the iraurite crucible to some extent prohibits the evaporation of fluoride. As the glass was melted in a silica crucible, Si was introduced due to the crucible corrosion. For the Raman spectra (Fig. 3d), the first broad peak in the low wavenumber region between 200–600 cm^−1^ is due to complex internal deformation bending modes of phosphate chains (both in chain PO2 and O-P-O bending). Peaks at ~706 cm^−1^ are assigned to the P-O-P symmetric stretching (Q^2^) mode of bridging oxygens between two phosphate tetrahedra, (POP)sym. The most intense peaks at ~1202 cm^−1^ indicate the symmetric resonances associated with the O-P-O non-bridging oxygens on Q^2^ phosphate tetrahedra, (PO2)sym. The small peak at ~1262 cm^−1^ that sits on the shoulder of the ~1202 cm^−1^ peak is the asymmetric stretching of non-bridging oxygens on Q^2^ phosphate tetrahedra, (PO2)asym. And no (PO3)sym stretching (Q^1^) mode or (PO4)sym stretching (Q^0^) mode of the P-O-P linkage which is supposed to be near 1030–1100 cm^−1^ and 990–1010 cm^−1^ respectively is observed in the Raman spectra^32^,^33^. The ^31^P NMR spectra (Fig. 3e) of the two types of 3ω transparent glasses further reveal their difference in the fine glass structure. The chemical shifts peaks near ~31 ppm, ~20 ppm and ~13 ppm are related to metaphosphate (Q^2^) tetrahedron^34^ and those near ~7 ppm and ~2 ppm are assigned to pyrophosphate (Q^2^) tetrahedron^35^.

**Figure 4:**
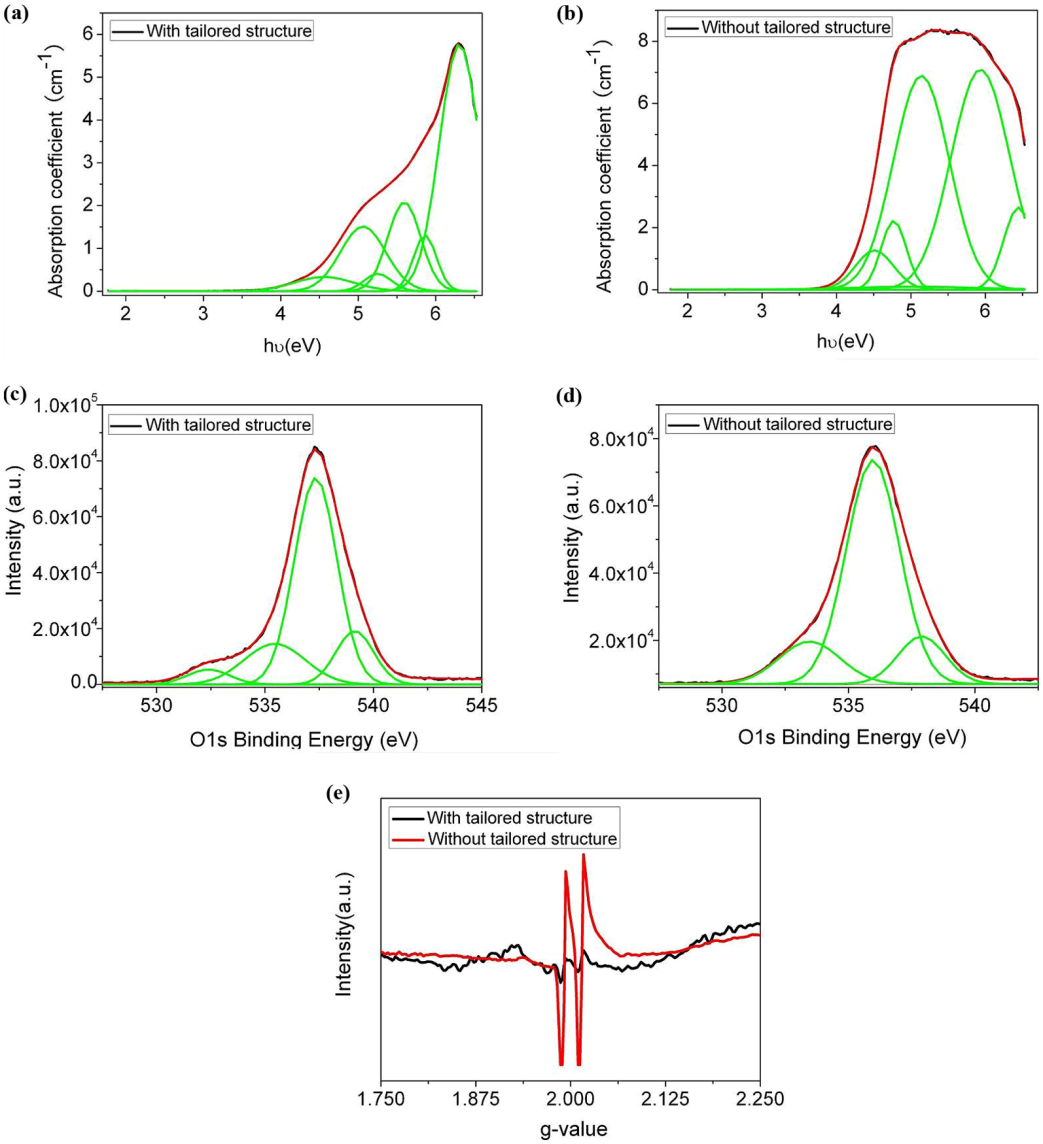
The absorption spectra, X-ray photoelectron spectra (XPS) and electron paramagnetic resonance (EPR) spectra for the 3ω transparent glasses with and without specially tailored structure: (a), (b) Absorption spectra with Gaussian peak fittings, (c), (d) O1s XPS spectra, and (e) EPR spectra of the 3ω transparent glasses after 3ω laser irradiations.

In further experiments, we have investigated their absorption spectra through Gaussian peak fitting method and ESR spectra, in order to detect the impurity or micro defects in these glasses, which are always the essential origin of a fluorescence emission. Fig. 4a and Fig. 4b present the Gaussian peak fitting of the absorption spectra for those two 3ω transparent glasses with and without specially tailored structure, respectively. The absorption peaks identified from the Gaussian peak fitting of the absorption spectra are shown in Table 2.

The O1s X-ray photoelectron spectra (XPS) collected from these two glasses are given in Fig. 4c and Fig. 4d. The XPS spectra were decomposed into a sum of Gaussian components in order to evaluate the bridging-to-terminal oxygen ratio in these two different glasses. The O1s spectra are best fitted with three or four Voigt peaks, indicating the bonding of oxygen atoms is complicated. The bands peaking at the lower binding energy (~531–535 eV) are assigned to the non-bridging oxygen (NBO) bonding to the glass modifier ions, and the peak at the higher binding energy (~536 eV) is related to bridging oxygen (BO) bonding to the glass former ions in the glass network^36^, representing distinct P = O, P-O-R^+^/R^2+^ and P-O-P, etc. In addition, the EPR spectra of these two 3ω transparent glasses after 3ω laser irradiation are measured (Fig. 4e). After laser irradiation, the 3ω transparent glass without tailored structure shows a typical character of POHC defects in EPR spectra^22^,^37^, which indicate the easier formation of POHC defects for this type of glass under the 3ω laser irradiations.

**Figure 5:**
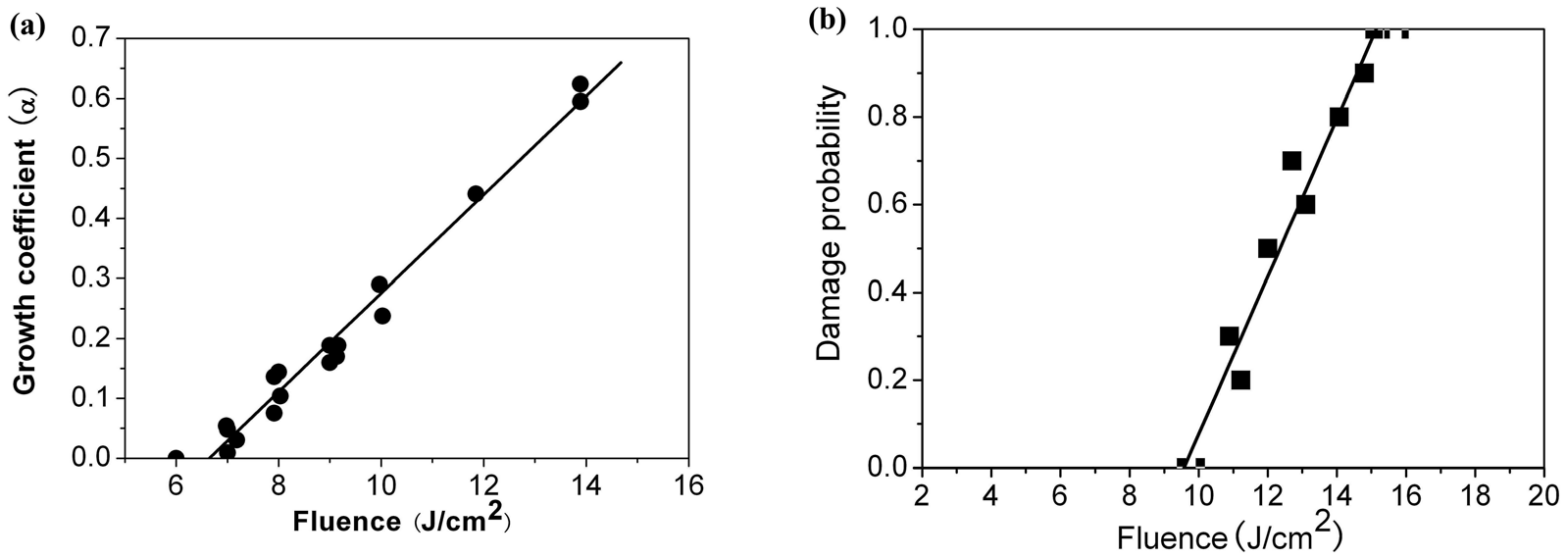
The growth damage and bulk damage properties of the fluoride-containing phosphate-based glass (3ω transparent glass): a) Measured exponential growth coefficient and b) Bulk damage probability as a function of beam fluence.

At last, we have further measured the growth damage and bulk damage properties of the fluoride-containing phosphate-based glass (3ω transparent glass), as shown in Fig. 5. In this stage we can only get a rough view of the growth damage property of the new phosphate-based glass, which is calculated to be about 6.5 J/cm^2^ (8 ns@3ω) through the x-intercept of plot of growth coefficients vs. beam fluence, as shown in Fig. 5a. The fitted slope of the data in Fig. 5a is about 0.075 per shot per J/cm^2^. The bulk damage threshold (8 ns@351 nm) of the 3ω transparent phosphate-based glass is measured to be about 9.6 J/cm^2^, as shown in Fig. 5b), which is lower than that of the fused silica (14.1 J/cm^2^) measured under similar conditions.

## Discussion

For the two absorptive samples, the 3ω internal transmittance decreases compared with that of the 3ω transparent glass, and their transmission edges in the UV spectral range shift both toward the long-wavelength. The red-shift of UV transmission edge is due to the doping of the absorptive ions in the absorptive glasses, i.e. Co^2+^ and Fe^2+^, which present different degrees of effect on the UV absorption and therefore make the internal transmittance at 351 nm variant. The trivalent ions of the 1ω absorptive ions, i.e. Fe^3+^ have obvious absorption in UV and near-UV range, but the 2ω absorptive ions (Co^2+^) don't^38^. Thus the 1ω absorptive glass shows a much larger red-shift value than the 2ω absorptive one.

However, the attention should also be paid that the 3ω absorptive coefficient will increase with higher doping levels of these absorptive ions. So a balance must be made between the 3ω internal transmittance and the specific absorptive coefficient at 1ω and 2ω laser wavelength. For example, with regard to the 1ω absorptive glass, the optical absorption at 1053 nm is due to Fe^2+^^25^,^26^. Therefore, the contrast result in Figure. 1d that 1ω absorptive glass's laser-induced damage thresholds at 1053 nm is lower than that of fused silica glass is attributed to its serious absorption of the 1053 nm laser light, which contributed to a more intense thermal damage^39^. Previous investigations on some aluminum potassium barium phosphate glasses indicate that the optical absorption at 1053 nm is due to the change of Fe^2+^/Fe^3+^ ratio in the glasses, which depends on a shift in the redox equilibrium between Fe^2+^ and Fe^3+^ that changes with the composition and the oxygen activity. The latter is related closely to the redox states of melting^27^, glass composition^25^, melting temperature^28^, oxidizing and reducing additives to the raw materials. Therefore, the balance between the internal transmission at 3ω and 1ω/2ω absorption coefficient can be realized through adjusting the doping concentration of absorptive ions in the glass batches and the control of the glass melting environment and other melting parameters.

With regard to the UV transmittance, as compared with the copper doped phosphate filter glasses^31^, here the investigated phosphate-based glasses show much higher 3ω internal transmittance and a blue-shift of the ultraviolet absorption edge, which is due to the introduction of fluorides and the reducing melting atmosphere that suppress the influence of Fe^3+^ impurity on the glasses' ultraviolet absorption. In addition to their featured absorption, the 1ω absorptive glass can also absorb little amount of the 2ω light, and the 2ω absorptive glass also shows much stronger absorption to the 1ω light. The features of ultraviolet (3ω) transmitting but intense absorption to 1ω and 2ω laser lights for these two phosphate-based glasses endow them with evident merits of selective absorption towards 1ω and 2ω laser wavelength light, respectively. If we can replace the color-separation grating optics in the FOA with the 1ω and 2ω absorptive optics, it is possible to filter the unconverted 1ω and 2ω lights clean after the 3ω laser light passes these two filter glasses.

The endowment of the selectively absorptive feature at 1ω and 2ω laser wavelength but meanwhile especially higher LIDT performance prior to the fused silica glass gives these two absorptive glasses potential high specific color phase separation ability by directly absorbing the 1ω or 2ω wavelength lights and therefore makes the 1ω and 2ω lights be separated from the 3ω laser light. Thus, using these new glass materials as color separation optics, just like the color separation gratings presently used in the US NIF^40^, it can also make the minimum NIF specifications satisfied that for transmitting >95% of 3ω light and <5% of 1ω and 2ω light, to the zero order. At the same time, to process the parallel-plate optics will not suffer so many processing difficulty compared with making the color separation gratings or silica wedge focus lens used in the US NIF.

For further analysis on the glass structure difference of the 3ω transparent glasses, firstly, the Raman signals measured for those two glasses (Fig. 3d) indicate their glass networks base mainly on Q^2^ tetrahedra, i.e. the basic structure for these 3ω transparent glasses can be described as a network of metaphosphate tetrahedral that are linked together through covalent bonding of oxygen atoms to form very long metaphosphate chains^32^,^33^. And the NMR spectra (Fig. 3e) in further suggest that both Q^2^ and Q^2^ phosphate related species are evident in these two 3ω transparent glasses. The most distinct difference is that the peak near ~31 ppm disappears for the 3ω transparent glass without tailored structure. This obvious decrease in shielding (less negative chemical shift) suggests a decreasing polymerization tendency of the glass network from the main component of metaphosphate tetrahedrons to pyrophosphate tetrahedrons to some extent. Therefore, the 3ω transparent glasses with tailored structure have relatively more densified glass network as compared with the ones without tailored structure.

Analyzed from the Gaussian peak fitting of the absorption spectra (Fig.4a and b), phosphor electron hole centers including PO_2_-EC, PO_3_-EC and PO_4_-EC are detected from both the 3ω transparent glass samples. Phosphorous oxygen bonded hole center (POHC) and Fe^3+^ related defects absorption^41^ are confirmed by the fitted absorption band at about 2.22 eV (558.8 nm) and 4.77 eV (260.0 nm), but only for the 3ω transparent glass without tailored structure (see Table 2). It is also regarded that the special reducing glass melting atmosphere helped decrease the POHC and Fe^3+^ related defects in the 3ω transparent glass, which directly results in the blue-shift of the ultraviolet absorption edge as indicated in Fig. 3b. On the other hand, the addition of polyvalent ions, e.g. Co^2+^ or Fe^2+^ can suppress the coloring effects due to the POHC formation, which absorb in the visible spectral range^41^. That is why the red-shift of the UV transmission edges for these two absorptive glasses is not so large.

From the XPS spectra in Fig. 4c and d, the ratio of the bridging oxygens to the total oxygens in the glasses, BO/(BO + NBO), that is calculated referring to the method by M. Karabulut *et al.*^36^, varies slightly. It decreases from 0.15 for the 3ω transparent glass with tailored structure to 0.13 for the one without tailored structure. This confirms the NMR analysis from another side that the 3ω transparent glass with tailored structure have more densified glass network due to the increase of bridged oxygens, compared with the one without tailored structure.

The 3ω transparent glass without tailored structure has more POHC and Fe^3+^ related defects but no visible fluorescence and is more prone to form POHC defects under the 351 nm laser irradiation, and the key point is that it has much lower LIDT value, as compared with the one with tailored structure. Thus, we can generally come to a conclusion by considering the above experimental facts that a notable laser-induced fluorescence results in a high LIDT value for the 3ω transparent glass, and the fluorescence band at ~776 nm does not originate from the POHC or Fe^3+^ related defects inside the glasses but should be closely related with the glass' special structure.

With regard to the laser-induced damage mechanism of these phosphate-based glasses, from present study results, we can come out a hypothesis that under the laser irradiations, the photons have direct interactions with these glasses to produce the original heat energy, the dense glass structure are not very easily to be broken apart under the laser irradiation, at the same time it can release part of the absorbed laser energy through the radioluminescence process, especially on the surfaces, thus to mitigate the direct deposited heat induced damage for these glasses, leading to the improvement of their resistance to the laser-induced surface damage. It needs more detailed work to analyze the contribution of the heat release by means of laser induced fluorescence among those heat mitigation methods.

It can be found that the growth damage threshold of the phosphate-based glass is larger than that of the fused silica^46^, which is about 5.0 J/cm^2^. However, the fitted slope of the data in Fig. 5a is as large as about 0.075 per shot per J/cm^2^. It is much higher than that of the fused silica^46^ that is between 0.03 and 0.05 per shot per J/cm^2^. It can be found that the exponential coefficients corresponding to a fluence of 8.0 J/cm^2^ are very close for the fluoride-containing phosphate-based glass and fused silica, under which the new phosphate-based glass maintains a relative smaller growth rate. However, its growth coefficient increases more rapidly as the beam fluence is larger than 8.0 J/cm^2^, and the value almost doubles as beam fluence increases from10.0 J/cm^2^ to 14 J/cm^2^. It suggests that at relative lower laser beam fluence the new phosphate-based glass has a comparable damage growth rate with that of the fused silica, and under larger beam fluence irradiations its damage grows faster than fused silica.

The bulk damage threshold (8 ns@351 nm) of the 3ω transparent phosphate-based glass is measured to be about 9.6 J/cm^2^, which is lower than that of the fused silica (14.1 J/cm^2^) measured under similar conditions. This may have close relationship with the laser-induced fluorescence observed in the bulk of these fluoride-containing phosphate-based glasses. It is thought that the effect of the radioluminescence process to release part of the absorbed laser energy and mitigate the direct deposited heat induced damage in the bulk of these glasses will become less important as compared with the condition of rear surface damage. On the contary, the influence of defects should be stressed. Gnerally, a luminescence phenomenon indicates the presence of some defects in the materials. These fluoride-containing phosphate-based glasses have multi-components including alkaline and alkali earth metals, thus sufficient P-O-R^+^/R^2+^ bonds will exist together with the P = O and P-O-P bonds in the glasses. The former's bonding strength is weaker compared with that of the latter ones, thus it is much weaker than that of the Si-O bonds in the fused silica glasses. Compared with the fused silica glass, the complex and non-perfect glass structure of these fluoride-containing phosphate-based glasses make them more liable to produce large amount of new defects in the bulk under the high dose of laser irradiation, which contributes to their lower bulk damage threshold. Besides, the researches on the other contributing factors, for instance the nonlinear refrective index and self-focusing, the mechnical and thermal properties of the glasses, etc, are underway.

In addition, under the same experimantal conditions, the bulk damage threshold of the 3ω transparent fluoride-containing phosphate-based glass is near 70% of that of the examined fused silica, but its surface damage threshold and growth damage threshold is higher than those of fused silica. By considering these factors comprehensively, we think the 3ω transparent fluoride-containing phosphate-based glass should have high resistance to nanosecond pulse laser-induced damage, which is better than or at least compariable with that of the fused silica under the same boundary conditions. Comprehensive test of these fluoride-containing phosphate-based glasses on the SG-III prototype system is under consideration.

Finally, even these phosphate-based glasses have shown some advantages over fused silica, it deserves attention that there still exist some drawbacks to overcome. Such as, the luminescence restricts them being used in other fields like UV microlithography, and they are difficult to manufacture in large aperture in industry. There is lots of work to be done. For example, to further increase their UV internal transmittance through improving the purity of raw materials, to confirm the interrelation between the laser-induced fluorescence with the glasses'microstructure and configurations in order to deplete the laser induced defects by means of the material structure engineering, and finally to regulate the glass composition and develop the appropriate continuous melting technology for producing the series of glasses in industry, etc. It demands many cooperative efforts before these types of fluoride-containing phosphate-based glass materials are developed to fulfill the requirements for engineering applications in ICF systems.

We have engineered a new series of ultraviolet transmitting, 1ω and 2ω absorptive fluoride-containing phosphate-based glasses with high pulse laser-induced surface damage thresholds. The 1ω and 2ω absorptive glasses are capable of separating third harmonic frequency from the fundamental and the second harmonic frequencies through selectively direct absorption, and their improved high damage threshold to the 351 nm nanosecond laser pulses, compared to standard fused silica glasses, is noteworthy. Use of these high performance glasses as color separation optics in ICF laser systems will be a radical departure from conventional color separation approaches by means of fused silica CSGs, thus novel designs of the FOA will be achieved. The studies on the relations between the laser-induced fluorescence, laser-induced damage threshold and the glass structure indicated that the laser induced fluorescence property is closely related to the glass structure, and it is a key factor that conduces to the high laser-induced damage resistance of the series of glasses.

## Methods

### Preparation of the fluoride-containing phosphate-based glasses

The undoped phosphate-based glasses (3ω transparent glasses) with little content of fluoride were prepared as references with high purity raw materials having a weight composition (wt%) of (0.5-2)Li_2_O-(3-5)K_2_O-(3-5)MgO-(7-10)BaO-(8-11)Al_2_O_3_-(59-64)P_2_O_5_-(0-1)YF_3_-(0-2)LaF_3_. The iron and cobalt ions as 1ω and 2ω absorptive agents are introduced into the 3ω transparent glasses to produce the 1ω and 2ω absorptive glasses, respectively. The series of fluoride-containing phosphate-based glasses were prepared in a 1.2 L iraurite crucible under reducing H_2_/N_2_ atmosphere to endow them with specially tailored material structure. In contrast, the 3ω transparent glass with same composition was intentionally made in an ambient air atmosphere in a 1.2 L silica crucible, which was named as the one without tailored structure, in order to compare its glass structure with that of the above glasses prepared in reducing atmosphere. For preparation of the samples, the mixed raw materials with each weight of 2600 g were melted in a 1.2 L iraurite crucible or silica crucible at 950°C. And the glass melts were refined and homogenized through mechanical stirring at 1200°C for 120 min and then cooled down to 980°C within 60 min. the homogenized glass melts were cast into a mold preheated at 300°C, then annealed at 400°C through a precision annealing process with a cooling rate of -0.2°C/h from 400°C to 300°C, followed by -1°C/h from 300°C to 150°C.

### Spectral characterization

Transmission spectroscopy measurements of the glass samples were carried out with a UV-VIS-NIR spectrophotometer (Shimadzu UV-3101) and the internal transmittance at investigated wavelength (τ_λ_) was achieved using two polished samples of the same glass but with different thickness (5 and 15 mm for each one) to compensate for Fresnel surface losses. Raman spectra were collected with a Jobin-Yvonne LabRam microscope with a 514 nm laser excitation in the range of 100–1600 cm^−1^. The ^31^P nuclear magnetic resonance (NMR) spectra were acquired at room temperature on Bruker MSL 360 and Chemagnetics CMX 240 spectrometers at 121.4 MHz using a 9.4 T magnet at typical spinning frequencies of 10 kHz. Analyses of the elemental composition in the two 3ù transparent glass samples with and without specially tailored structure were carried out by means of the energy dispersive spectrometer (EDS). The X-ray photoelectron spectroscopy measurements were conducted on a Thermo Advantage X-ray photoelectron spectrometer (XPS) at room temperature using Al K_α_ (1486.6 eV) as the radiation source. The acceleration voltage was 12 kV, and the emission current was 10 mA. Electron paramagnetic resonance (EPR) spectra were recorded at 100 K using a Bruker EMX spectrometer operating in the X-band frequency (9.677 GHz) with field modulation 100 kHz. The microwave power used was 20 mW.

### Laser-induced damage thresholds and laser-induced fluorescence measurements

The precision annealing glass samples were cut into 25 mm × 25 mm × 7 mm for the surface LIDT measurements and precisely polished using zirconia (ZrO_2_) micro-particles. Here nonuse of cerium polishing power (CeO_2_) is because it has large influence on the laser-induced damage at 351 nm due to its strong absorption of the ultraviolet light^42^,^43^. After precision polishing, the glass samples were measured to have a parallelism less than 1′, a surface flatness with <λ/6 at 632.8 nm in the 90% area of central aperture and high surface quality with 20–10 scratch-dig. The surface roughness (R_q_) of these samples was about 0.8 nm. All the samples used for the LIDT measurements had no any coating in both surfaces.

1-on-1 laser induced surface damage studies of these investigated glasses were carried out using a Q-switched Nd-glass pulse laser according to ISO 11254-1^44^, which provided the test beam of 351 nm, 527 nm and 1053 nm with a pulse width at half maximum of 8–12 ns. The laser beam of 1053 nm was first generated at a small aperture master oscillator stage and amplified to several joules using Nd doped laser glass. The fundamental frequency light was then converted to its second harmonic (527 nm) and third harmonic (351 nm) lights through the frequency converters (KDP crystals). The incident beam energy was measured in proportion by a calibrated energy probe (Ophir PE50BF) before the test sample with a silica wedge. The laser energy ratio of sample to power meter was 26.3. The peak energy density fluctuation was <5%. A lens with the focus length of 1498 mm was used to focus the laser on the rear surface of the measured samples. The profile of the pump beam is near-Gaussian with 1/e^2^ diameter of about 4.12 mm, 3.63 mm and 2.56 mm at 1053 nm, 527 nm and 351 nm on the rear surface of the glass sample. The damage of the measured samples was diagnosed by a real time observation system composed of a 3 megapixel CCD camera and a set of long working distance objectives in dark field mode. The damage was defined to be any visible change in the laser irradiated site by measurement of the scattering light under the dark field. Per fluence, at least ten laser shots were tested at separate sites. Repeat this procedure for other pulse energies to develop a plot of damage probability versus laser fluence. Linear extrapolation of the damage probability data to zero damage probability yields the threshold laser fluence for each sample. Fused silica samples that have the same thickness (25 mm × 25 mm × 7 mm) and similar surface polishing level were used as reference material under similar excitation conditions.

The damage growth on the rear surface of the 3ω transparent glass was parameterized by the growth threshold (*Fth*) and the measured growth coefficient (α)^45^. The growth coefficient was obtained through an exponential growth fitting of the damage area as a function of the beam fluence (*F*)^46^. The laser damage was initiated at 351 nm with a single shot at laser pulse fluence levels of about 25 J/cm^2^ and the increase in damage area was measured in terms of gray scaling due to subsequent shots at lower fluence.

The bulk damage threshold of the 3ω transparent glass and the fused silica were measured using a Q-switched Nd-YAG laser with a 9.3 ns FWHM Gaussian pulse at 355 nm by means of an R-on-1 measuring mode. The pump beam is near-Gaussian with 1/e^2^ diameter of about 13 mm after the spatial filtering. The precisely polished samples with size of 50 mm × 50 mm × 20 mm were used. The laser beam was focused with a lens of 70-cm focal length and the focal plane was 26 cm apart from the rear surface of the sample. Change in the laser irradiated site in the bulk near the rear surface was observed by a CCD for determination of the bulk damage in the testes samples.

The laser-induced luminescence phenomenon was captured synchronously during the LIDT experiments by a digital panchromatic camera (30 frames per second) and a Phantom v9.1 high speed digital camera (500 frames per second), respectively. The laser-induced fluorescence spectra were investigated by setting a time-resolved fiber spectrometer (ANDOR TECHNOLOGY SR-303i) aside the tested samples perpendicular to the laser beam propagation path and recorded with a CCD (DU-897E-CS0-#BV) from a side view. A high-bandpass filter (at 400 nm) was set up in the front of the fiber spectrometer to avoid the incoming light of the 351 nm laser beam. The details of the experimental setup of the LIDT and LIF testing system referred to our previous research^47^.

## Figures and Tables

**Table 1:** Comparative analyses of the elemental composition in the two 3ω transparent glass samples with and without specially tailored structure by means of the energy dispersive spectrometer (EDS)

Glass	O	F	P	K	Mg	Ba	Al	Y	La	Si
3ω glass(tailored)	45.98	4.88	15.35	3.73	0.61	9.27	2.01	16.17	2.01	0
3ω glass(nontailored)	47.49	3.36	14.07	3.80	0.48	9.68	2.24	16.39	1.90	0.59

**Table 2:** Absorption peaks identified from the Gaussian peak fitting of the absorption spectra for the two 3ω transparent glasses with and without specially tailored structure

Glass	Phosphor electron hole centers					Phosphor oxygen bonded hole center	Impurity related defects
	PO_2_-EC (eV)	PO_3_-EC (eV)		PO_4_-EC (eV)		POHC (eV)	Fe^3+^ (eV)
3ω glass (tailored)	4.56	5.86	5.60	5.25	5.06	-	-
3ω glass (nontailored)	4.51	5.94	-	5.14	5.04	2.22	4.77
